# Supplementary material for: Statin Use Decreases the Incidence of Hepatocellular Carcinoma: An Updated Meta-Analysis
Source: Cancers (Basel). 2020 Apr 3;12(4):874. doi: 10.3390/cancers12040874 (PMC7225931; doi:10.3390/cancers12040874)
Supplement: Supplementary file 1 [file cancers-12-00874-s001.pdf]

Table S1. Risk of bias assessment and quality of included studies.

Observational Studies[a]				
	**Selection**	**Comparability**	**Outcome**	**Overall quality**
**Björkhem-Bergman 2014**	***	**	**	H
Butt 2015	***	**	**	H
Chang 2017	***	**	**	H
Chen 2014	***	**	**	H
El-Serag 2009	***	**	**	H
Friedman 2016	*	*	*	L
German 2019	**	**	**	H
Goh 2019	**	**	**	H
Hsiang 2015	*	*	*	L
Kaplan 2019	***	**	**	H
Kim 2018	**	**	**	H
King 2013	*	*	*	L
Lai 2013	***	**	**	H
McGlynn 2014	***	**	**	H
McGlynn 2015	***	**	**	H
Mohanty 2016	*	*	*	L
Simon 2019	**	**	**	H
Tran 2019	**	**	**	H
Tsan 2012	**	**	**	H
Tsan 2013	**	**	**	H
Marelli 2011	**	*	*	L
Friis 2005	**	**	**	H

Randomized Controlled trials [b]								
	**1**	**2**	**3**	**4**	**5**	**6**	**7**	
Sato 2006	L	L	L	L	L	L	L	H
Matsushita 2010	L	L	L	L	L	L	L	H
Emberson 2012	L	L	L	L	L	L	L	H

L, low; H, high; U, unclear; M, moderate.

[a] Study quality assessment performed by means of Newcastle/Ottawa scale (each asterisk represents if the respective criterion within the subsection was satisfied)

[b] Cochrane Collaboration’s tool for assessing the risk of bias across 7 domains: 1 (Random sequence generation), 2 (Allocation concealment), 3 (Blinding of participants and personnel), 4 (Blinding of outcome assessment), 5 (Incomplete outcome data), 6 (Selective reporting) and 7 (Other bias).
